# The association between low back pain and sleep quality in adults 60 years and older: a systematic review

**DOI:** 10.1186/s12998-026-00656-w

**Published:** 2026-06-24

**Authors:** Pegah Rahbar, Jessica J. Wong, Astrid DeSouza, Efrosini Papaconstantinou, Dan Wang, Sheilah Hogg-Johnson, Pierre Côté

**Affiliations:** 1 Department of Graduate Studies, Canadian Memorial Chiropractic College, Toronto, ON Canada; 2 Institute for Disability and Rehabilitation Research, Ontario Tech University, Oshawa, ON Canada; 3 Institute for Health Policy, Management, and Evaluation, University of Toronto, Toronto, ON Canada; 4 Faculty of Health Sciences, Ontario Tech University, Oshawa, ON Canada; 5 School of Physical Therapy, Faculty of Health Sciences, Western University, London, ON Canada; 6 Institute for Clinical Evaluation Sciences, Western University, London, ON Canada; 7 Department of Epidemiology and Biostatistics, Schulich School of Medicine & Dentistry, Western University, London, ON Canada; 8 Department of Research and Innovation, Canadian Memorial Chiropractic College, Toronto, ON Canada

**Keywords:** Sleep quality, Low back pain, Older adults, Systematic review

## Abstract

Poor sleep quality is a health concern in older adults, with low back pain (LBP) as a hypothesized risk factor. We aimed to synthesize evidence on the association between LBP and sleep quality among adults ≥ 60 years. We conducted a systematic review, registered protocol (Open Science framework #u7bc4) and reported it according to PRISMA. We searched MEDLINE, Embase, CINAHL, and PsycINFO (inception to December 2025). We included cross-sectional, cohort, and case–control studies on LBP and sleep quality among adults ≥ 60 years. We utilized a combination of OpenAI’s ChatGPT and fluent members in our team to translate non-English studies after which we screened and assess their quality. We synthesized evidence from low and moderate risk of bias studies. Our search retrieved 2394 articles. After removing duplicates, we screened 2237 citations for eligibility, with 191 studies eligible for full-text screening. 13 studies were relevant and appraised. Our synthesis includes five studies of moderate to low risk of bias (three cross-sectional and two cohort studies). Although measures of LBP and sleep quality varied across the included studies, all demonstrated an overall positive small association between the two constructs. Chronic LBP is positively associated with poor sleep quality in aging adults. Our study highlights methodological limitations in existing research and calls for more rigorous epidemiological studies.

**Supplementary Information:**

The online version contains supplementary material available at 10.1186/s12998-026-00656-w.

## Introduction

Poor sleep quality is among one of the health concerns in the aging population. In 2020, 47.6% of Canadian adults 65 years and older reported fair or poor sleep quality and 55% met the recommendation of 7–8 h of sleep per day [1, 2]. Although aging is characterized by some levels of normal decline in sleep duration and maintenance (mostly attributed to reduction in melatonin hormone), higher levels of decline in sleep quality can be problematic for this population [3]. Poor sleep quality has been associated with a number of negative health outcomes including diabetes, cardiovascular disorders, depression, dementia, obesity, and mortality [4, 5] Little is known about the etiology of poor sleep quality in the aging population. One hypothesized risk factor for poor sleep quality is the presence of pain [4].

Previous systematic reviews have reported an association between chronic spinal pain and the quality, satisfaction, duration, efficiency, and latency of sleep among adults 18 years and older [6, 7] with no stratified analysis to investigate this association for older adults ≥ 60 years. However, little is known about the association between Low Back Pain (LBP) and sleep quality in older adults (≥ 60 years old). This is significant because LBP is the leading cause of years lived with disability in the world [8]. The prevalence of LBP increases with age up to 80 years old and the prevalence of disability related to LBP peaks between the ages of 80–85 years [8]. LBP affected 619 million individuals globally in 2020 and is estimated to affect 843 million by 2050, mostly due to population growth and aging [8]. Finally, compared to younger adults, adults 65 years and older are more likely to be severely impacted by LBP[8].

To our knowledge, no previously published systematic reviews have investigated the association between LBP and sleep quality among adults aged 60 years and older. Given the global burden of LBP and poor sleep quality in the aging population, investigation of this association among older adults is important. Despite some evidence of an association between spinal pain and sleep quality in the general adult population, it remains unclear whether this relationship remains the same in adults aged 60 years and older. Therefore, we conducted a systematic review of the literature to synthesize the evidence on the association between LBP and sleep quality among adults 60 years and older.

## Methods

We registered our protocol with Open Science Framework (https://osf.io/u7bc4). We reported our systematic review using the Preferred Reporting Items for Systematic Review and Meta-Analyses (PRISMA) statement [9]. We chose to conduct a systematic review as opposed to other review studies to provide the current critically appraised evidence on this topic.

### Eligibility criteria

Studies that made the following criteria were considered to be eligible: 1) study of the association of acute or chronic LBP and sleep quality; 2) cross-sectional, case–control, or cohort studies published in a peer-reviewed journal; 3) no language restriction; and 4) investigated adults aged ≥ 60 years.

### Population: adults 60 and older

Our target population was adults aged ≥ 60 years. We included this age group based on the United Nations’ definition of older adults [10]. We excluded any studies that included adults 59 years old or younger from this review unless studies provide stratified results for older adults.

### Exposure: low back pain

LBP is defined as pain between the 12th rib and the inferior gluteal folds with or without referred/radicular pain in one or both legs [11]. We included acute (less than 12 weeks [12]), chronic LBP, or recurrent pain that lasts for 12 weeks or more in which the pain intensity may vary over time [12, 13]. We excluded studies with LBP due to major structural or pathological etiology such as fractures, dislocations, infections, malignancies, neoplasms, and inflammatory arthropathies [12, 13].

### Outcome: sleep quality

Sleep quality is “the individual’s self-satisfaction with all aspects of sleep” [14]. Indicators of sleep quality include sleep efficiency, sleep latency, sleep duration and wake after sleep onset [14]. We included studies that investigated at least one sleep quality indicator. Specifically,

- Sleep efficiency: the ratio of the total time asleep over the total time spent in bed [14].
- Sleep latency: time it takes a person to go from the state of wakefulness to sleep [14].
- Sleep duration: total amount of sleep minus any arousals at night [14].
- Wake after sleep onset: total amount of wake time after sleep onset until the final awakening [14].

Studies investigating self-reported sleep measures such as interviews, surveys, sleep diaries, and sleep-related outcome measures, or objective measures by utilizing polysomnography, actigraphy, and functional MRIs, etc. were included. However, we excluded studies that focused on sleep-related diagnoses including but not limited to sleep apnea, insomnia, and restless leg syndrome.

### Information sources and search strategy

We searched MEDLINE, Embase, CINAHL, and PsycINFO from inception to December 18, 2025. The search strategy was developed in collaboration with an experienced health sciences librarian (see Appendix A for MEDLINE strategy). The search strategy was reviewed by a second librarian following the Peer Review of Electronic Search Strategies (PRESS) guideline [15]. Search terms included keywords and subject headings (MeSH) for each of the databases with the three key concepts of older adults, low back pain, and sleep (see Appendix A).

### Study selection

Two independent reviewers screened the titles and abstracts and categorized them as “possibly relevant” or “irrelevant” based on our inclusion/exclusion criteria. Two independent reviewers screened the full text of the studies deemed possibly relevant and categorized them as relevant or irrelevant; and documented the reasons for exclusion. Any disagreements during each phase of screening were resolved by discussion between the paired reviewers to reach a consensus. If consensus was not reached, a third reviewer was consulted. We contacted study authors, if additional information was required to screen the studies.

Reviewers were first trained on a random sample of 50 titles and abstracts, and 25 full-text articles. If the percent agreement was less than 80% at each pilot phase, the team discussed to clarify the eligibility criteria before commencing the screening.

### Risk of bias appraisal

We used the JBI checklists for cross-sectional studies, cohort studies and case–control studies [16, 17] to assess the quality of eligible studies. These checklists focus on the evaluation of selection bias, measurement bias, confounding, and allow for an overall assessment of how bias impacted the internal validity of a study. We used both prevalence and analytical cross-sectional JBI checklists to assess the quality of the cross-sectional studies reporting both prevalence and an analysis of associations. Two reviewers independently appraised the relevant articles and categorized the articles as low, moderate, or high risk of bias. Studies were classified as high risk of bias if they demonstrated major potential sources of selection, measurement, or confounding bias, as indicated by one or more “No” responses to the relevant JBI appraisal items. Studies with no major concerns but with a limited number of “Unclear” responses were rated as having a moderate risk of bias. Any disagreements were resolved by a third independent reviewer.

Prior to formally starting the critical appraisal of studies, we trained reviewers on using the critical appraisal tools on a sample of five eligible articles. Any discrepancies were discussed between the reviewers, and if needed, resolved by a third reviewer. An agreement of 80% was required before formally starting the critical appraisal of studies. We contacted study authors if, additional information was required to critically appraise the studies. One eligible study was authored by our team and was therefore reviewed and appraised by two independent reviewers who were not involved in the original work or this systematic review (see Acknowledgements).

### Data items and data collection process

We extracted the following data: authors, year of publication, location/setting of the study, study design, participant demographics, number of participants, LBP measures, sleep measures, and measure of crude and adjusted association between LBP and sleep (e.g. odds ratios, relative risks, prevalence ratios). If there were missing data, the authors of the study were contacted. One reviewer extracted data from the low and moderate risk of bias studies. An independent second reviewer checked the extracted data for accuracy. If the agreement was less than 80% for all items in the table, then the team discussed to clarify each item and consulted with the third reviewer to reach a consensus.

### Data analysis and evidence synthesis

We calculated the percent agreement for the two phases of screening, data extraction and ratings of the critical appraisal. We used the Synthesis Without Meta-Analysis (SWiM) framework to guide our narrative synthesis of the low and moderate risk of bias studies [18]. We reported associations between LBP and sleep quality (and its various elements) as odds ratios (OR), relative risks (RR), prevalence ratios (PR) and correlations with 95% confidence intervals (CI). We calculated OR, PR, RR, and their 95% CI if a study did not report a measure of association but provided sufficient data to allow for computation.

## Protocol deviation

We report two protocol deviations. First, we originally planned to include studies published in English or French. However, we identified possibly relevant studies in other languages and adapted our method to include these studies. Specifically, we invited two members at our institute who were fluent in Portuguese and Chinese to review these articles and assess their eligibility. Moreover, we translated studies published in other languages (i.e. Danish, Norwegian, Spanish) using OpenAI’s ChatGPT (November 2024 version) and screened their full text for eligibility and reached consensus. The lead author translated and extracted data (if needed) which was reviewed by members fluent in Chinese and Portuguese languages to ensure the accuracy of the translations. We did not have members fluent in other languages to verify the other translations. Second, we originally planned for two reviewers to independently extract data. However, due to lack of time and resources, we modified our method to have one reviewer extracting the data and a second reviewer to review the accuracy of the extracted information.

## Results

### Study selection

**Fig. 1:**
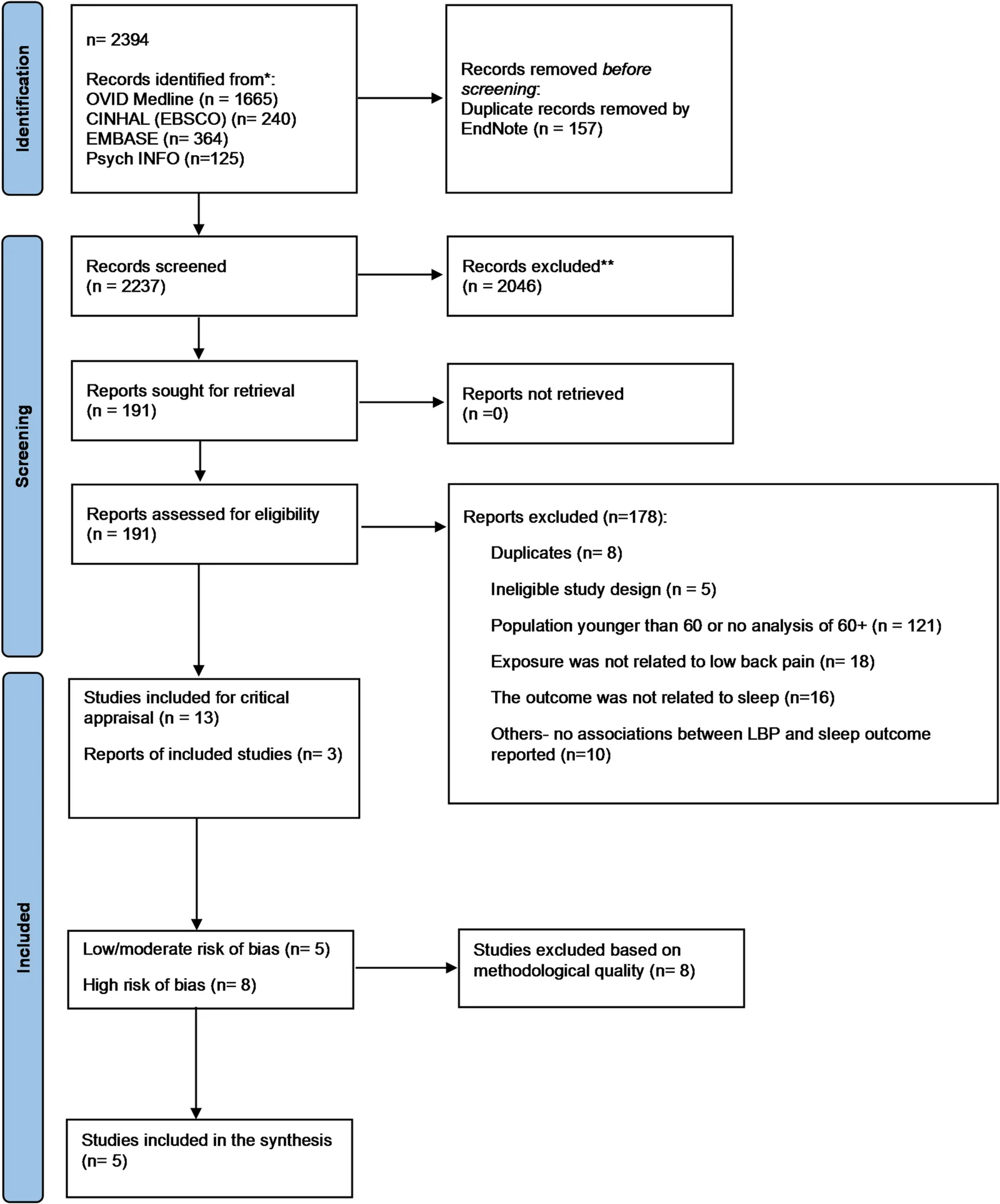
Preferred Reporting Items for Systematic reviews and Meta‑Analyses flow diagram for study selection

Our search retrieved 2394 articles. We removed 157 duplicates and screened the eligibility of 2237 title and abstracts (Fig. 1). Of the 191 full texts that were possibly relevant, 13 were eligible for critical appraisal [20–30]. The two main reasons for exclusion during full text screening were ineligible population (n = 121) and ineligible exposure (n = 18) (Fig. 1).

The percent agreement for the pilot phase of title/abstract and full text screening were 98% and 92%, respectively, while the agreement in formal phases for title/abstract and full text screenings were 97.8% and 98.9%, respectively. The agreement for pilot phase of critical appraisal and the full critical appraisal phase was 60% (3/5 studies) and 75% (6/8 studies), respectively. For disagreements, the two reviewers reached a consensus after a discussion. For data extraction phase, there was a 94.2% (33/35 items) agreement between the reviewers with minimal suggestions to improve the data presentation from the second reviewer.

### Risk of bias

Of the 13 studies included, three were rated a low risk [19–21], two were rated a moderate risk [22, 23] and eight studies were rated as high risk of bias [24–31] (Tables 1, 2, 3). The eight high risk of bias studies had methodological limitations regarding sampling methods [25–30], inclusion/exclusion criteria [25], response rates [24–28, 30, 31], and unclarity about consideration of confounding variables [25–30] (Tables 1, 2, 3). We contacted the authors to seek clarification regarding uncertainties in their studies. Only one author responded [31], indicating that they had no further information about non-responders, while the others did not provide a reply.

**Table 1:** Risk of Bias Assessment for Eligible Prevalence Studies using the JBI Checklist for Prevalence Studies

Authors, Years	1.1	1.2	1.3	1.4	1.5	1.6	1.7	1.8	1.9	Overall risk of bias
Blay, 2007 [19]	Y	Y	Y	Y	Y	Y	Y	Y	Y	Low
Li, 2010 [20]	Y	Y	Y	Y	Y	Y	Y	Y	Y	Low
Chen, 2011[24]	Y	Y	U	Y	Y	Y	Y	Y	N	High
Efendioglu, 2022 [25]	Y	U	U	U	U	Y	Y	Y	U	High
Grimby,1997 [26]	Y	U	Y	Y	Y	Y	Y	Y	N	High
Hosseini, 2018 [27]	Y	N	Y	Y	U	U	U	Y	U	High
Wiliamson, 2020 [31]	Y	Y	Y	Y	Y	Y	Y	Y	N	High
Rahbar, 2025 [23]	Y	Y	Y	Y	Y	Y	U	Y	U	Moderate

*Y,* Yes; *N*, No; *U*, Unclear; 1.1 Sample frame: Was the sample frame appropriate to address the target population?; 1.2 Sample recruitment: Were study participants recruited in an appropriate way?; 1.3 Sample size: Was the sample size adequate?; 1.4 Study setting/Setting: Were the study subjects and setting described in detail?; 1.5 Coverage of sample: Was data analysis conducted with sufficient coverage of the identified sample?; 1.6 Identification of the condition: Were valid methods used for the identification of the condition?; 1.7 Condition measured: Was the condition measured in a standard, reliable way for all participants?; 1.8 Statistical Analysis: Was there appropriate statistical analysis?; 1.9 Response rate: Was the response rate adequate, and if not, was the low response rate managed appropriately?

**Table 2:** Risk of Bias Assessment for Eligible Cross-sectional Analytic Studies using the JBI Checklist

Authors, Years	1.1	1.2	1.3	1.4	1.5	1.6	1.7	1.8	Overall risk of bias
Blay, 2007 [19]	Y	Y	Y	Y	Y	Y	Y	Y	Low
Li, 2010 [20]	Y	Y	Y	Y	Y	Y	Y	Y	Low
Chen, 2011[24]	Y	Y	Y	Y	Y	Y	Y	Y	High
Efendioglu, 2022 [25]	U	Y	Y	Y	N	N	Y	Y	High
Grimby, 1997 [26]	U	U	Y	U	N	N	U	U	High
Hosseini, 2018 [27]	Y	Y	U	U	U	U	U	Y	High
Inoue, 2023 [28]	Y	U	Y	Y	N	N	Y	Y	High
Rudy, 2007 [30]	Y	U	Y	Y	U	U	Y	U	High
Wiliamson, 2020 [31]	Y	Y	Y	Y	Y	Y	Y	Y	High
Rahbar, 2025 [23]	Y	Y	U	Y	Y	Y	U	U	Moderate

*Y,* Yes; *N*, No; *U*, Unclear; 1.1 Inclusion criteria: Were the criteria for inclusion in the sample clearly defined?; 1.2 Subjects and setting: Were the study subjects and the setting described in detail?; 1.3 Measurement of exposure: Was the exposure measured in a valid and reliable way?; 1.4 Criteria for condition: Were objective, standard criteria used for measurement of the condition?; 1.5 Confounding factors: Were confounding factors identified?; 1.6 Strategies for Confounding: Were strategies to deal with confounding factors stated?; 1.7 Outcome measurement: Were the outcomes measured in a valid and reliable way?; 1.8 Statistical analysis: Was appropriate statistical analysis used?

**Table 3:** Risk of Bias Assessment for Eligible Cohort Studies using the JBI Checklist

Authors, Years	1.1	1.2	1.3	1.4	1.5	1.6	1.7	1.8	1.9	1.10	1.11	Overall risk of bias
Jacobs, 2006 [29]	Y	Y	U	N	N	N	Y	Y	Y	Y	Y	High
Morelhão, 2021[21]	Y	Y	Y	Y	Y	Y	Y	Y	Y	Y	Y	Low
Lee, 2025 [22]	U	Y	U	Y	Y	Y	U	Y	U	U	Y	Moderate

*Y,* Yes; *N*, No; *U*, Unclear; 1.1 Were the two groups similar and recruited from the same population?; 1.2 Were the exposures measured similarly to assign people to both exposed and unexposed groups?; 1.3 Was the exposure measured in a valid and reliable way?; 1.4 Were confounding factors identified?; 1.5 Were strategies to deal with confounding factors stated?; 1.6 Were the groups/participants free of the outcome at the start of the study (or at the moment of exposure)?; 1.7 Were the outcomes measured in a valid and reliable way?; 1.8 Was the follow up time reported and sufficient to be long enough for outcomes to occur?; 1.9 Was follow up complete, and if not, were the reasons to loss to follow up described and explored?; 1.10 Were strategies to address incomplete follow up utilized?; 1.11 Was appropriate statistical analysis used?

The three low risk of bias studies had strengths in describing sampling frames, sampling methods, setting, sufficient sample coverage, considering some confounders, and appropriate statistical analysis [19–21]. Two studies with moderate risk of bias had limitations with measurement of low back pain and sleep quality as the validity and reliability those questions were unclear [22, 23].

### Study characteristics

Among the five articles included, three were cross-sectional [19, 20] and two were cohort studies [21] (Table 4). While all the included studies were in English, one cross-sectional study [20] was written in Chinese which was reviewed by two Chinese fluent reviewers independently and translated by Open AI’s ChatGPT (November 2024 version) by the lead author for additional review.

### Low back pain

The definition of LBP varied among all five studies. The three cross-sectional studies reported chronic back pain of six months [19], chronic back problems expected to last or lasted six months and is diagnosed by a health professional [23] and chronic LBP of undefined duration that is diagnosed in a hospital or any healthcare setting above township level (i.e. primary care setting) [20]. One cohort study defined chronic LBP over the past three months, localized between the costal margin and the gluteal fold with or without lower limbs radiation, while measuring pain intensity levels using numeric rating scales (NRS) [21]. Another cohort study assessed back pain in the past four months, including frequent back pain (≥ 3 consecutive reports in one year), and severe/activity limiting back pain in the past four months that reduced usual activities [22].

### Sleep quality

Sleep quality was predominantly measured with self-reported questions. One cross-sectional study [20] and one cohort study [21] assessed sleep quality using Pittsburg Sleep Quality Index (PSQI). This 21-item questionnaire evaluates sleep quality in the past month through seven domains of subjective quality of sleep, sleep latency, sleep duration, sleep efficiency, sleep disturbances, use of medication to sleep, and diurnal dysfunction [21]. Another cross-sectional study assessed disturbed sleep in the past 30 days using one sleep item in the Short Psychiatric Evaluation Schedule (SPES) [19]. In one cross-sectional study, sleep quality was characterized as three outcomes of sleep duration, trouble falling or staying asleep, and trouble having a refreshing sleep [23]. In another cohort study [22], sleep problems were assessed using self-reported questions and actigraphy which were used to derive an overall sleep health score (see Table 4).

### Cross-sectional studies

Three cross-sectional studies reported a positive association between chronic LBP and poor sleep quality. One study (n = 6963) suggested that participants with chronic back pain were more likely to report disturbed sleep (crude PR = 1.40 [95% CI 1.37 to 1.43]) [19]. Another study (n = 1680) reported an adjusted OR = 1.17 [95% CI 1.12 to 2.50] for the association between self-reported diagnosed chronic LBP and poor sleep quality measured by PSQI [20]. The third study (n = 9814, weighted n = 2,378,212) reported a positive association between chronic back problems and not meeting the recommended sleep duration guidelines, having trouble falling or staying asleep, and having an unrefreshed sleep (adjusted PR = 1.19 [95% CI 1.11 to 1.27], adjusted PR = 1.54 [95% CI 1.32 to 1.79], adjusted PR = 1.45 [95% CI 1.20 to 1.79], respectively; see Table 4) [23].

### Cohort studies

One cohort study (n = 215) reported that chronic LBP intensity measured by NRS was associated with higher PSQI scores (representing worse sleep quality) at six-month follow-up (β = 0.14 [95% CI 0.01 to 0.26]) [21]. This means that with one point increase in NRS, there was an average of 0.14-point increase in the PSQI score which represent a minimal change [21]. Additionally, the study reported relatively similar associations between higher PSQI scores at baseline and higher LBP intensity at 6-months (β = 0.18 [95% CI 0.07 to 0.30]) [21].

Another cohort study (n = 963) reported that any report of back pain (at least once per year), frequent back pain (more than three reports per year), severe and activity limiting back pain overall preceded sleep problems in a 6-year follow up (OR = 1.12 [95% CI 1.03 to 1.21], OR = 1.17 [95% CI 1.01 to 1.38], OR = 1.19 [95% CI 1.02 to 1.38], and OR = 1.25 [95% CI 1.05 to 1.48], respectively; see Table 4) [22].

**Table 4:** Summary of evidence from three low risk of bias studies on association of low back pain and sleep quality among adults 60 years and older

Authors, Years, Country	Setting	Sample demographics, (N)	Time to follow up	Low back pain definition	Sleep quality definition	Measures of association
Cross sectional studies						
Blay et al., 2007, Brazil[19]	Face-to-face survey through interviews (1995–1996), in Rio Grande do Sul., Brazil	Community-dwelling, non-institutional population of adults ≥ 60 yrs, (N = 6963)	N/A ‡	Back pain experienced at any time in the past 6 months	Subjective measure of sleep quality from one item of SPES: In the last 30 days “Is your sleep disturbed?” (Yes/No)	Prevalence of back pain among those with disturbed sleep vs. those without back pain:
		Age groups: 60–70 56.7% Female 66% Caucasian 84.2% Rural 65% Married 45.4% Education < 4 yrs 84.6%		Positive back pain was assigned if individuals reported any form of treatment whether it included outpatient or inpatient physician contact or other kinds of treatment		No back pain [ref] Crude PR ** = 1.40 [95% CI †: 1.37, 1.43] Logistic regression between disturbed sleep and back pain, adjusting for sociodemographic factors
Li et al., 2010, China [20]	Face-to-face surveys conducted in 6 townships from 3 cities of Anhui province in China (Sept-Nov 2009)	Adults 60 and older (N = 1680) 68.44 ± 7.10 years Male 50.1% Education below elementary 88.5% Occupation Farming 89.5% Married 68.2%	N/A	Chronic LBP that is diagnosed by the hospital at or above township level	PSQI including sleep quality, sleep duration, sleep disturbance, sleep medications, and daytime dysfunction < 5 Good sleep quality 5–7 general sleep quality > 7 poor sleep quality	Logistic regression comparing good vs. poor sleep quality Adjusted for sociodemographic, marital status, lifestyle, and chronic diseases No back pain [Ref] OR = 1.17 [1.12, 2.50]
Rahbar et al., 2025 [23]	Canadian Community Health Survey (CCHS) 2015–2016, Ontario, Canada	Ontarian adults ≥ 60 yrs, excluding individuals living in reserves and other Indigenous settlements in the provinces, full-time members of Canadian forces, institutionalized population, and those living in Région du Nunavik and Région des Terres-Cries-de-la-Baie-James, (N = 9814, weighted N = 2,378,212) Age: 59% 60- 69 yrs Sex: 53.4% Female	N/A	Chronic back problems expected to last or lasted 6 months or more, diagnosed by a health professional	1. Number of hours spent sleeping each night: “How long do you usually spend sleeping each night?”	Modified Poisson Regression for association between chronic back problems and three sleep quality outcomes. Adjusted for demographic, socioeconomic, living arrangements, marital status, chronic health conditions, and lifestyle behaviours
		Main activity: 66.0% Retired		“Do you have back problems, excluding scoliosis, fibromyalgia, and arthritis?” (Yes, No)	“7 to less than 9 h” (meeting recommended sleep duration) versus “less than 7 h or 9 or more hours”	Crude and adjusted PR** [95% CI†]:
		Highest level of education: 57.3% Post secondary education			(not meeting recommended sleep duration)	No chronic back problems [ref]
		Marital status: 65.6% Married			2. Frequency of trouble going to sleep or staying asleep: “How often do you have trouble going to sleep or staying asleep?”	Sleeping outside of recommendations per night (< 7 or > 9 h): cPR: 1.28 [1.20, 1.38] aPR: 1.19 [1.11, 1.27]
		Total household income: 36.8% ≥ $80,000			“all the time and most of the times” versus	Having trouble going to sleep or staying asleep all the time or most of the time cPR: 1.92 [1.63, 2.27] aPR: 1.54 [1.32, 1.79]
		Perceived general health: 52.3% very good to excellent			“sometimes, rarely, and never”	Rarely or never having a refreshing sleep cPR: 2.10 [1.73, 2.51] aPR: 1.45 [1.20, 1.79]
		Diabetes: 16.7% yes			3. Frequency of refreshing sleep: “How often do you find your sleep refreshing?”	
		High blood pressure: 41.77% yes			“all the time and most of the times” versus “sometimes, rarely, and never	
		Mood disorders: 6.89% yes				
		Anxiety disorders: 5.15% yes				
		Non-smoker: 88.39% yes				
		Non-smoker: 88.39% yes				

Authors, Years, Country	Setting		Sample demographics, (N)		Time to follow up		Low back pain definition		Sleep quality definition		Measures of association	
Cohort studies												
Morehlão et al., 2021, Brazil[21]		At home baseline interview, 15-min telephone interviews at follow up (Mar 2017- Dec 2018)		Community-dwelling adults ≥ 60 yrs, LBP for at least 3 months. Excluded subacute or acute LBP. (N = 215)		6 months		LBP: Localized pain or discomfort between the costal margin and the gluteal fold with or without radiation to the lower limbs. “Do you have LBP today?” If yes, have you had LBP in the past 3 months?”		Sleep quality in the past month (PSQI)		Multivariable linear regression model adjusting for age, BMI, comorbidities, and depression
				Mean age: 71.0 ± 7.5				Average pain intensity in the past 24 h: 11-point NRS		Scores 0–21		LBP related NRS and sleep quality measured by PSQI:
				Mean BMI: 28.0 ± 5.0						Good sleep quality 6 or less points/ Poor 7–12 points/ Insomnia more than 12 points)		β = 0.14 [0.01, 0.26]. With one score increase in NRS, the PSQI score increased by 0.14
				Women 77.7%								
				Married 54.4%								
				Mean PSQI score: 10.3 ± 3.2								
				Mean NRS: 4.5 ± 3.2								
Lee et al., 2025 [22]		Osteoporotic Fractures in Men Study (MrOS), a prospective cohort study of older men (≥ 65 years), 2000–2012, United States		Men, ≥ 65 years, from six geographically dispersed clinical centers (Birmingham, AL; Minneapolis, MN; Palo Alto, CA; Pittsburgh, PA; Portland, OR; San Diego, CA), Able to walk independently with or without aid, Not have a bilateral hip replacement (N = 963)		6 years		Surveyed every 4 months		Two sleep visits:		An autoregressive cross-lagged panel model (CLPM) to estimate bidirectional prospective associations between a composite sleep problems score and several dichotomized back pain measures. Adjusted for sociodemographics, body mass index, smoking, alcohol, physical activity, chronic conditions, depressive symptoms, cognitive function, fall history, and relevant medication use.
				Age at sleep visit 1, mean (SD): 74.53 (4.6)				“Have you experienced any back pain in the past four months?” (Yes/No)		Sleep questionnaire (perceived sleep quality and sleepiness)		β [95% CI†]: Any back pain, No [ref]
				Ethnicity, non-Hispanic white: 88.47%				Having any back pain: any report of back pain in the survey in 1 year		Actigraphy on nondominant wrist for a minimum of five consecutive 24-hr periods following the visit		Sleep problems: 0.11 [0.03, 0.19] Exp(β) = 1.12
				Education, college graduate: 61.68%				Frequent back pain: three consecutive reports of back pain		Sleep diaries (bedtime, wake times, when actigraphy was removed)		Irregular sleep: -0.01 [-0.04, 0.03]
				Marital status, currently married: 87.64%				Severe back pain/ activity limiting back pain: “In the past four months, how many days did you have severe back pain?” and “In the past four months, how many days did you cut down on things you usually do because of back pain?” (0-9; >2 defined as having severe/activity limiting back pain)		Ru-SATED dimensions of sleep health measures:		Sleep dissatisfaction: 0.10 [0.07, 0.14] Exp(β) = 1.11*
				Body mass index, mean (SD): 27.2 (3.72)						Sleep regularity, timing, efficiency, and duration dimensions measured by actigraphy		Poor daytime alertness: 0.03 [-0.01, 0.05]
				Smoking, yes: 57.42%						Satisfaction and alertness measured by self-report questionnaires		Suboptimal sleep timing: 0.03 [-0.01, 0.06]
				Alcohol intake, 0 drink/week: 30.32%						Sleep irregularity (greater than 1 SD of average sleep midpoint [the time halfway between the sleep onset and the sleep offset]),		Insufficient sleep: -0.03 [-0.05, 0.01]
				Physical activity (PASE score), mean (SD): 157.36 (68.71)						Sleep dissatisfaction (either bad/very bad sleep quality or having sleep disturbances at least once a week over the past month from the Pittsburgh Sleep Quality Index)		Suboptimal sleep duration: -0.01 [-0.04, 0.03] Frequent back pain, No [ref]
				Number of chronic conditions, mean (SD): 0.8 (0.83)						Poor daytime alertness (over the past month, measured by Epworth Sleepiness Scale, >10)		Sleep problems: 0.16 [0.01, 0.32] Exp(β) = 1.17
				Depressive symptoms (GSD-15), minimal depressive symptoms (≤ 2): 82.87%						Suboptimal sleep timing (average sleep midpoint ≤2 a.m. or >4 a.m.)		Irregular sleep: 0.01 [-0.06, 0.07]
				Cognitive function (3MS, 0–100), mean (SD): 94.09 (4.71)						Inefficient sleep (wake after sleep onset ≥90 min/night; with supplemental analyses using sleep efficiency <85%, defined as total sleep time divided by time in bed)		Sleep dissatisfaction: 0.18 [0.12, 0.25] Exp(β) =1.20*
				Fall history, yes: 26.38%						Suboptimal sleep duration (<6 hr or >9 hr)		Poor daytime alertness: 0.04 [-0.02, 0.09]
				Medication use: Antidepressants 6.75%, Benzodiazepine 3.53%, Sedatives 1.87%, sleep medication 10.28%						Sleep problems:		Suboptimal sleep timing: 0.03 [-0.04, 0.09]
				Sleep problem composite score (0–6), mean (SD): 1.47 (1.20)						Summed the scores of six binary indicators (0 = not having the condition, 1 = having the poor sleep condition), and a higher score represented more sleep problems (0–6).		Insufficient sleep: -0.05 [-0.11, 0.01]
												Suboptimal sleep duration: -0.01 [-0.08, 0.05]
												Severe back pain, No [ref]
												Sleep problems: 0.17 [0.02, 0.32] Exp(β) = 1.19
												Irregular sleep: -0.03 [-0.07, 0.02]
												Sleep dissatisfaction: 0.12 [0.05, 0.20] Exp(β) = 1.27*
												Poor daytime alertness: 0.02 [-0.03, 0.07]
												Suboptimal sleep timing: 0.06 [0.01, 0.12] Exp(β) =1.06*
												Insufficient sleep: 0.04 [-0.03, 0.10]
												Suboptimal sleep duration: -0.02 [-0.08, 0.05]
												Activity limiting back pain, No [ref]
												Sleep problems: 0.22 [0.05, 0.39] Exp(β) = 1.25
												Irregular sleep: -0.01 [-0.06, 0.05]
												Sleep dissatisfaction: 0.13 [0.06, 0.19] Exp(β) =1.14*
												Poor daytime alertness: 0.01 [-0.05, 0.07]
												Suboptimal sleep timing: 0.09 [0.03, 0.16] Exp(β) = 1.09*
												Insufficient sleep: 0.03 [-0.04, 0.10]
												Suboptimal sleep duration: -0.02 [-0.09, 0.06]

^**^ PR = Prevalence ratio; OR = Odds ratio * Calculated by the review team † CI = Confidence interval

^‡^ N/A no data given, not applicable N: sample size; SD: Standard deviation

SPES: Short Psychiatric Evaluation Schedule; LBP: Low back pain; PSQI: Pittsburgh Sleep Quality Index; BMI: Body Mass Index; NRS: Numeric Rating Scale; PASE: Physical Activity Scale for the Elderly; GSD: Geriatric Depression Scale; 3MS: Modified Mini Mental State

## Discussion

To our knowledge this is the first systematic review investigating the association between LBP and sleep quality specifically among adults aged 60 years and older. Our synthesis includes five moderate to high quality studies that consistently found that chronic LBP is positively associated with poor sleep quality. Yet, it is important to note that the magnitude of this association was overall small after considering some of the important covariates.

Overall, the research on how LBP is associated with poor sleep quality is limited [7, 21]. Although we did not identify any systematic reviews in this topic, focused specifically on older adults, our findings align with patterns reported in systematic reviews of adult populations. In our search of studies of adult populations, findings have been mixed with some studies reporting a positive association between chronic LBP and poor sleep quality [32, 33], while others have found no significant associations [34, 35].

The included studies in our synthesis were heterogenous in their definitions and measurement of both LBP and sleep quality. Almost all the studies only relied on self-reported sleep measures, which, although common in epidemiological research, may contribute to variability in findings. Most of the studies assessed sleep using a single measure (e.g. PSQI total score or single-item sleep question). In contrast, one cross-sectional study examined the association between LBP and multiple sleep quality indicators including sleep duration, trouble falling or staying asleep and unrefreshing sleep [23]. Notably, this study reported larger effect estimates, particularly for trouble falling or staying asleep. These findings may suggest that examining specific indicators of sleep quality, rather than relying solely on an overall sleep score, may provide additional insight into the association between LBP and poor sleep quality.

While causality cannot be established from cross-sectional studies, the positive associations they identified were supported by findings from two included cohort studies. One high quality cohort study examined the bidirectional association between LBP and sleep quality over a 6-month follow up and reported β estimates that were small and similar in magnitude [21]. In contrast, a second cohort study with a 6-year follow up suggested a potential temporal relationship, indicating that LBP may precede poor sleep quality among males aged 65 years and older [22].

## Strengths and limitations

Our study has several strengths. We described our protocol a priori and registered it on the Open Science Framework. We used the PRISMA and the SWiM checklists to report our synthesis and used the JBI checklists to appraise the quality of the eligible studies. We did not restrict our search to specific languages to ensure we captured all relevant studies. Finally, we only synthesized the findings of low to moderate risk of bias studies which improves the certainty of our evidence synthesis.

Our review has limitations. First, we did not include grey literature in our review so we could not assess potential publication bias. Second, we were unable to confirm the accuracy of the translated material from languages other than Chinese and Portuguese as we did not have anyone fluent in those languages on our team. Therefore, we might have excluded five articles in other languages at the screening stage that may have been potentially relevant (see Appendix B). Third, we applied a relatively broad screening criterion for the definition of exposure and the outcome (i.e. LBP and sleep quality), as most studies do not provide a clear definition. While this approach may introduce heterogeneity in the exposure and outcome classification, it reflects the need for standardized definition of LBP and sleep quality across the literature. Additionally, almost all included studies utilized subjective sleep measures, hence our synthesis is solely based on self-reported measure of sleep quality which may not fully capture the physiological sleep measures.

## Implications and future research

Our findings are mainly from cross-sectional studies indicating the need for future high-quality studies to further investigate this association and to establish causality. We excluded eight eligible studies due to their methodological limitations which highlights the need for improving epidemiological methods in this field. Researchers should consider using reporting checklists such as Strengthening and Reporting of Observational Studies in Epidemiology (STROBE) to ensure a clear description of their studies [36]. A clear description of study sample, sampling methods, demographics, potential confounding and covariate factors as well as a clear definition of LBP and sleep quality will strengthen the methodological rigor of the studies in this field. Researchers should also consider describing the validity of the tools used to measure LBP and sleep quality and conduct a non-responder analysis in case of low response rates. Although the use of objective sleep measures (e.g., polysomnography) may be limited in large epidemiological settings due to cost and feasibility constraints, future studies incorporating objective assessments could help clarify whether comparable associations are present.

Our findings highlight the positive association between LBP and poor sleep quality among adults 60 and older, suggesting that sleep health may be a relevant consideration in LBP management (and vice versa). Clinicians may be mindful of the association of LBP with sleep quality and consider strategies to assess and address poor sleep quality as part of a comprehensive approach to caring for older adults with LBP.

## Conclusion

We found moderate to high-quality evidence that chronic LBP is positively associated with poor sleep quality in older adults ≥ 60 years, though current literature indicates a small association. We recommend adopting standardized, clearly defined measures of LBP and sleep quality, along with validated sleep assessment tools, to strengthen the quality of evidence in this field. Given the global burden of both LBP and poor sleep quality among older adults and the role of various demographic, socioeconomic, and health related factors in this association, it is also important to investigate this association further in cross-sectional and cohort studies that account for a wider range of confounders. Future studies should examine multiple sleep indicators (e.g. short versus long sleep duration, latency, maintenance) rather than relying on a single measure when investigating the relationship between LBP and sleep quality.

## Supplementary Information

Below is the link to the electronic supplementary material.

Supplementary Material 1

Supplementary Material 2

## Data Availability

Data sharing not applicable to this article as no datasets were generated or analyzed during the current study.
